# Population Structure and Genetic Diversity Within the Endangered Species *Pityopsis ruthii* (Asteraceae)

**DOI:** 10.3389/fpls.2018.00943

**Published:** 2018-07-11

**Authors:** E. Anne Hatmaker, Margaret E. Staton, Adam J. Dattilo, Ðenita Hadziabdic, Timothy A. Rinehart, Edward E. Schilling, Robert N. Trigiano, Phillip A. Wadl

**Affiliations:** ^1^Department of Entomology and Plant Pathology, The University of Tennessee, Knoxville, Knoxville, TN, United States; ^2^Biological Compliance, Tennessee Valley Authority, Knoxville, TN, United States; ^3^Thad Cochran Southern Horticultural Laboratory, United States Department of Agriculture-Agricultural Research Service, Poplarville, MS, United States; ^4^Department of Ecology & Evolutionary Biology, The University of Tennessee, Knoxville, Knoxville, TN, United States; ^5^United States Vegetable Laboratory, United States Department of Agriculture-Agricultural Research Service, Charleston, SC, United States

**Keywords:** endemic, federally endangered, genetic structure, microsatellites, Ruth’s golden aster

## Abstract

*Pityopsis ruthii* (Ruth’s golden aster) is a federally endangered herbaceous perennial endemic to the Hiwassee and Ocoee Rivers in southeastern Tennessee, United States. Comprehensive genetic studies providing novel information to conservationists for preservation of the species are lacking. Genetic variation and gene flow were evaluated for 814 individuals from 33 discrete locations using polymorphic microsatellites: seven chloroplast and twelve nuclear. A total of 198 alleles were detected with the nuclear loci and 79 alleles with the chloroplast loci. Gene flow was estimated, with the Hiwassee River (*N*_m_ = 2.16; *F*_ST_ = 0.15) showing higher levels of gene flow and lower levels of population differentiation than the Ocoee River (*N*_m_ = 1.28; *F*_ST_ = 0.19). Population structure was examined using Bayesian cluster analyses. Nuclear and chloroplast analyses were incongruent. From the chloroplast microsatellites, three clusters were identified; all were present in sampling sites at both rivers, indicating a lack of allele fixation along rivers. Nuclear markers revealed two clusters and separated by river. When the Hiwassee River locations were analyzed, four clusters were identified for both the chloroplast and nuclear microsatellites, though the individuals clustered differently. Analysis of the Ocoee River revealed two clusters for the chloroplast microsatellites and three for the nuclear microsatellites. We recommend *P. ruthii* be managed as four populations for the Hiwassee River and three populations for the Ocoee River. Our results provide critical genetic information for *P. ruthii* that can be used for species management decisions to drive future population augmentation/reintroduction and ex situ conservation efforts.

## Introduction

Endangered species generally have small or declining populations, and often these populations suffer from inbreeding and erosion of genetic diversity resulting in elevated extinction risks (Frankham, 2003). The delineation of conservation units is a critical first step in plant conservation to ensure that resource managers know what constitutes a population. The federally endangered *Pityopsis ruthii* (Small) Small, also known as Ruth’s golden aster, is endemic to southeastern Tennessee, United States, where it grows on unshaded phyllite rock boulders in and on the adjacent slopes of the Hiwassee and Ocoee Rivers (Bowers, 1972). The two geographically separated rivers are the only populations of *P. ruthii* (2n = 2x = 18) that remain in the wild, one containing approximately 1,000 individuals along ∼3 km of the Ocoee River and a larger population of around 12,000 individuals along ∼6.5 km of the Hiwassee River (Moore et al., 2016). The riparian habitat of *P. ruthii* is highly dynamic and is typified by seasonally high temperatures, frequent drought, and regular inundating flood flows. Thomson and Schwartz (2006) noted that altered river flow due to damming has led to higher competition rates and lower seed dispersal, which has put *P. ruthii* at risk for short-term extinction.

In spite of its endangered status due to its small population size and narrow geographic range, relatively little research has focused on species recovery until recently (Moore et al., 2016). Studies on genetic diversity have been limited prior to the development of microsatellite markers for *P. ruthii* (Wadl et al., 2011a). Notably, *in vitro* (Wadl et al., 2011b) as well as vegetative (Wadl et al., 2014) propagation methods for *P. ruthii* were developed to enable reintroduction and augmentation studies (Wadl et al., 2018). The U.S. Fish and Wildlife Service (USFWS) identified actions necessary for recovery, including defining what constitutes a viable population and developing management protocols that ensure the existence of self-sustaining populations along both rivers (USFWS, 1992, 2012). Therefore, knowledge of population structure and delineation of conservation units for *P. ruthii* are critical to long-term conservation and management efforts.

Estimating genetic diversity and population dynamics for *P. ruthii* will provide a framework to inform ecological and conservation issues for species management. This study also enables discourse on broader issues such as the role of damming on the genetic diversity of riparian species, which can be monitored over time using molecular means outlined. Community ecology has been shown to change with damming, often leading to an increase in non-native plants, which can drastically alter the composition of the riparian ecosystem (Greet et al., 2013). The relatively small system, extensive sampling, and annual census of individuals, combined with the knowledge of all known populations of *P. ruthii* (Moore et al., 2016) provides an ideal scenario to conduct comprehensive population studies as a model for other endangered plant species. *Pityopsis ruthii* can therefore serve as a model plant to explore the effects of conservation techniques and river flow on a riparian plant.

Information of genetic structure is essential for understanding the scales over which dispersal, genetic drift, and selection operate in populations (Slatkin, 1987). Genetic research determining population diversity can be invaluable when forming and revising an endangered species management plan, as maintaining diversity is critical for conservation. Fragmentation or disappearance of natural populations can lead to reduced gene flow among populations; this in turn increases genetic differentiation among populations and genetic structuring due to genetic drift (Ouborg et al., 2006). The effects of isolated and fragmented populations on attributes of the genetic structure of *P. ruthii*, in particular genetic erosion, are unknown, especially when habitat degradation is taken into account. Here we test our null hypothesis that individuals along each river constitute a population and that further genetic structuring will not be supported by observations. Our alternative hypothesis is that further genetic structuring will reveal more precise population parameters, with the possibility of multiple populations along each river. We define a population as a large group of individuals that exchange genetic material. For the purpose of clarity, each discrete location sampled for this study will be referred to as a sampling site. Estimating genetic diversity and genetic drift, as well as determining gene flow of the endangered plant species *P. ruthii* using chloroplast and nuclear microsatellites will advance molecular ecology and conservation efforts for the species. Understanding genetic drift and gene flow allows further inferences about the history of the species and delineation of viable populations, as called for by the USFWS.

Neutral, non-coding, and easy to develop, microsatellites are suitable when studying small occurrences of native plants under a variety of evolutionary pressures, such as in *P. ruthii*. Microsatellite markers in particular are a popular and cost-effective way to measure genetic diversity within populations. The short tandem repeats in non-coding sections of DNA exhibit co-dominant inheritance in nuclear DNA and are highly variable (Wan et al., 2004). This study uses both chloroplast and nuclear microsatellites to examine genetic diversity within *P. ruthii*. Nuclear microsatellite markers were developed and demonstrated to be suitable for determination of genetic diversity in a limited sample size from the Hiwassee and Ocoee River populations of *P. ruthii* (Wadl et al., 2011a). Additionally, microsatellite markers were developed from the chloroplast genome and used as a complement to the nuclear markers to understand the natural history of the species as well as recent ecological changes. The haploid nature of the chloroplast allows identification of loci with a single allele in each individual, creating a nice complement for use with biparentally inherited molecular markers such as nuclear microsatellites. Chloroplast microsatellites have uniparental inheritance, a lack of recombination, and a slower mutation rate than nuclear microsatellites (Provan et al., 2001). Chloroplast microsatellites are especially useful for understudied groups such as native plants with a lack of *a priori* knowledge about genetic structure and species of little economic importance (Wheeler et al., 2014). This study is the first to use highly variable microsatellite loci to examine the genetic diversity and population structure of *P. ruthii*. The results of this study provide valuable genetic information that can be combined with the development of effective propagation and reintroduction techniques (Wadl et al., 2011b, 2014), geospatial mapping of all known occurrences, and stressors affecting *P. ruthii* (Moore et al., 2016) to guide conservation and management decisions for the species.

## Materials and Methods

### Plant Materials and Microsatellite Genotyping

In 2010, leaf samples were collected from 814 *P. ruthii* individuals across 33 sampling sites: 25 on the Hiwassee River and 8 on the Ocoee River (**Supplementary Data Sheet S1**). Sampling sites were established as groups of plants based on well delineated breaks in suitable habitat within the riparian area for each river. The average census counts (2011–2014) for the sampling sites sampled ranged from 15 to 1034 plants for the Hiwassee River and 12–491 plants for the Ocoee River (Moore et al., 2016). At the time of sampling, we collected leaf tissue from all individual plants at a known sampling site if the total number of individuals was less than 50. When sampling sites were greater than 50, we used a random number generator to randomly sample up to 50 individuals. Since sampling occurred in 2010, additional locations have been discovered and further delineation of the locations has occurred based on natural breaks in suitable habitat (Moore et al., 2016), explaining why some sampling sites have fewer than five individuals sampled. Lastly, sampling of an individual is difficult because *P. ruthii* reproduces asexually by rhizomes and the extent of spread via crevices is unknown; therefore, distinguishing individual plants is challenging or nearly impossible. To ensure sampling of individuals rather than clones, plants occupying the same crevice and occurring less than 15 cm apart were considered an individual, whereas plants more than 15 cm apart in the same crack and plants occupying apparently distinct crevices were considered separate individuals. We further attempted to ensure that a single genetic individual was used for microsatellite genotyping by using DNA isolated from a single leaf for each individual.

Total genomic DNA was isolated from leaf samples using the Qiagen DNeasy Plant Mini Kit (Qiagen, Valencia, CA, United States) according to the manufacturer’s protocol. The concentration and quality of the DNA was measured with a NanoDrop ND-1000 spectrophotometer (NanoDrop Technologies, Inc., Wilmington, DE, United States). Twelve polymorphic microsatellite primer pairs for *P. ruthii* (Wadl et al., 2011a) were used to genotype individuals. Additionally, a 400 base pair (bp) genomic DNA library was developed from a single *P. ruthii* genotype and sequenced using the Ion Torrent Personal Genome Machine (ThermoFisher, Waltham, MA, United States). The de novo assembly yielded chloroplast genome reads that were then assembled and screened for microsatellites using the program Imperfect SSR Finder (Stieneke and Eujayl, 2007). Twenty-one loci were obtained and screened with samples from five locations along the Hiwassee and Ocoee Rivers. Using a 2% agarose gel and the Qiaxcel Advanced Capillary Electrophoresis System (Qiagen, Valencia, CA, United States), 7 loci were found to be polymorphic among a subset of 66 individuals, 11 from each of 6 sampling sites—2 from the Ocoee River and 4 from the Hiwassee River.

For microsatellite analyses, *P. ruthii* individuals were amplified using a 10 μl polymerase chain reaction with 4 ng genomic DNA, 2.5 mM MgCl_2_, 1X GeneAmp PCR Buffer (Applied Biosystems, Carlsbad, CA, United States), 0.2 mM dNTPs, 0.25 μM primer (forward and reverse), 0.4 U AmpliTaq Gold DNA polymerase (Applied Biosystems), 5% dimethyl sulfoxide (DMSO), and sterile water. The reaction was run using the following cycling conditions: 94°C for 3 min; 35 cycles at 94°C for 40 s, 55°C for 40 s, and 72°C for 30 s; and one cycle at 72°C. All individuals were amplified using 12 nuclear microsatellite loci and 7 chloroplast microsatellite loci. Amplicons were visualized using the QIAxcel Advanced Capillary Electrophoresis System (Qiagen) and sized using an internal 25–500 bp DNA size marker. Electropherograms were visualized using the software BioCalculator (Qiagen) for nuclear data and the next iteration of the software, ScreenGel version 1.4.0 (Qiagen) for chloroplast data. The raw allelic data was compiled into an Excel worksheet and all nineteen loci were binned using the Excel add-in FLEXIBIN (Amos et al., 2007), which bins raw allele length data into allele size categories using an automated algorithm and reduces false inflation of diversity. The binned data was used in subsequent data analyses (**Supplementary Data Sheet S1**). When both datasets were combined for Bayesian analyses, chloroplast data was coded as diploid rather than haploid, as STRUCTURE does not allow mixed ploidy data.

### Analysis of Nuclear Microsatellites

The Excel add-in GenAlEx 6.5 (Peakall and Smouse, 2012) and Arlequin 3.5 (Excoffier and Lischer, 2010) was used to estimate genetic diversity and calculate diversity indices among the 814 samples, including the mean number of alleles (*N*_A_), effective number of alleles (*N*_E_), observed (*H*_O_) and expected (*H*_E_) heterozygosity, gene flow (*N*_m_), and *F* statistics across all populations for each locus. The program HP-Rare (Kalinowski, 2005) was used to calculate allelic richness (*A*_R_). A Bayesian analysis was performed using STRUCTURE version 2.3.4 (Pritchard et al., 2000) using the admixture model, which infers whether the individual *i* has inherited a portion of its genetic material from ancestors in population *k*. For measuring different values of *k*, 20 independent replicates were made for each *k* value between 1 and 10 for the Hiwassee and Ocoee River populations separated, and *k* = 1–30 for the combined nuclear data. A burn-in period of 250,000 iterations and 250,000 Markov Chain Monte Carlo (MCMC) repetitions were used in all analyses. Estimation of the best *k* value was determined using STRUCTURE Harvester (Earl and von Holdt, 2012), which identifies the appropriate number of clusters (*k*) using the *ad hoc* statistic Δ*k*. This is based on the second order rate of change in the log probability of the data between successive values of *k*. The analysis was performed three times: once with all individuals, once with individuals found along the Hiwassee River, and once with individuals found along the Ocoee River. Additionally, subsampling of populations over 25 individuals was performed using a random number generator for further Bayesian analyses, to ensure accuracy despite uneven sampling. Subsampling yielded a smaller dataset of 683 individuals.

The apportionment of genetic variation was determined by an analysis of molecular variance (AMOVA) using ARLEQUIN (Excoffier et al., 2005). The significances of variance components for each hierarchical comparison (among populations, among individuals, among individuals within populations) were tested using 99,999 permutations. GenAlEX 6.5 (Peakall and Smouse, 2012) was used for pairwise calculations of *F*_ST_ and gene flow estimates between populations. To determine the occurrence of isolation by distance (IBD), a Mantel test between the genetic and geographic distances was evaluated using GenAlEx with 9,999 permutations.

BOTTLENECK version 1.2.02 (Piry et al., 1999) was used to determine which populations may have undergone significant reductions in size and to test for allele frequency mode-shifts (i.e., distortion away from the typical L-shaped distribution). We also tested for the presence of an excess of observed heterozygotes by using the Wilcoxon signed rank test to evaluate deviations from 50:50 deficiency/excess (Luikart and Cornuet, 1998). Heterozygote excess was tested under all three mutation models, infinite alleles (IAM), two-phase (TPM), and the step-wise mutation model (SMM). For TPM we set *p*s = 0.9 (the frequency of single step mutations) and the variance of those mutations as 12. These values are typical for many microsatellite markers.

### Analysis of Chloroplast Microsatellites

The Excel add-in GenAlEx 6.5 (Peakall and Smouse, 2012) was used to calculate several diversity indicators. The haploid data set was combined with geographic coordinates for input into the program. Population differentiation (*F*_ST_) was calculated for all samples, and Shannon’s diversity index, diversity (*h*), and unbiased diversity (*uh*) were calculated for each population and locus. Nei’s gene diversity (Nei, 1973) was calculated for populations using GenAlEx as well. A principal coordinates analysis (PCA) based on a covariance matrix was also calculated. A Mantel test using population pairwise geographic distance and genetic distance (Mantel, 1967) was performed to determine isolation by distance.

Clustering of the populations was performed using STRUCTURE 2.3.4 (Pritchard et al., 2000). Posterior probabilities were estimated for three different chloroplast data sets: all sampling sites, Hiwassee River sampling sites, and Ocoee River sampling sites. When the data was separated by river, *k* = 1–10. When all chloroplast data was analyzed together, *k* = 1–30. An admixture model was assumed for all analyses. The burn-in generation and the MCMC were set to 250,000, with 20 iterations. Delta K, the optimal number of clusters for the sample set, was estimated using the Evanno method (Evanno et al., 2005) through STRUCTURE Harvester, as described previously. We also subsampled the dataset for the chloroplast data using the same 683 individuals as the subsampled nuclear dataset, with no more than 25 individuals per location. Analysis of the subsampled data set was conducted using 100,000 burn-in iterations and 50,000 MCMC, as outlined by Puechmaille (2016).

A standard AMOVA was calculated using the program ARLEQUIN 3.5 (Excoffier and Lischer, 2010), using a pairwise distance matrix with 99,999 permutations and a threshold of 5% missing data, which excluded 10 individuals. Three hierarchical AMOVA analyses were performed for both the nuclear and the chloroplast data sets. The first analysis included all sampling sites as one hierarchical group, the second analysis included all sampling sites on the Hiwassee River, and the third included all sampling sites from the Ocoee River. Haplotype frequency was analyzed using the program ARLEQUIN.

## Results

### Genetic Diversity in Nuclear Microsatellites

A total of 814 *Pityopsis ruthii* individuals from 33 sampling sites were genotyped using 12 nuclear and 7 chloroplast microsatellite loci (**Supplementary Data Sheet S1**). For the 12 polymorphic nuclear loci, 198 alleles were detected (**Supplementary Table S1**). Loci demonstrated an overall departure from HWE due to significant heterozygote deficiency when all 814 samples were analyzed together. The number of alleles detected per locus (*A*) ranged from 9 (PR028) to 24 (PR029), with a mean allelic richness (*A*_R_) of 1.80, ranging from 1.68 (PR002) to 1.89 (PR029 and PR031). The observed heterozygosity (*H*_O_) was 0.49 and deviated from the expected heterozygosity (*H*_E_) of 0.65. Population differentiation was large (*F*_ST_ = 0.24) and the inbreeding coefficient was moderate (*F*_IS_ = 0.22). Average gene flow (*N*_m_) across loci was 0.90 and ranged from 0.45 (PR009) to 1.76 (PR035).

The genetic variability of the 12 microsatellite loci was assessed for each sampling site and between the two rivers (**Table 1**). For individuals from the Hiwassee River sampling sites, mean allelic richness was 1.99 and ranged from 1.52 (H-02-01) to 4.22 (H-12-06), whereas among the Ocoee River individuals, mean allelic richness was 1.56 and ranged from 1.49 (O-04-01) to 1.60 (O-06-01). On the Hiwassee River observed heterozygosity (*H*_O_) was 0.53 and ranged from 0.38 (H-03-01) to 0.71 (H-09-02) whereas expected heterozygosity (*H*_E_) was 0.64 and ranged from 0.51 (H-02-01) to 0.74 (H-04-01). Across the Ocoee River sampling sites, *H*_O_ was 0.36 and ranged from 0.32 (O-03-01) to 0.40 (O-02-01) while *H*_E_ was 0.54 and ranged from 0.46 (O-04-01) to 0.59 (O-06-01). The inbreeding coefficient was higher for the Ocoee River individuals (0.35) than the Hiwassee River individuals (0.22) and the range of inbreeding coefficient values was much larger for the Hiwassee River individuals (0.22 to –0.01) compared to the Ocoee River individuals (0.27–0.44).

**Table 1:** Genetic variability of 12 nuclear microsatellite loci estimated for 33 sampling sites of *Pityopsis ruthii*.

River	Sampling site	Sample size	*A*_R_	*H*_O_	*H*_E_	*F*_IS_	Private alleles
Ocoee	O-06-01	49	1.60	0.38	0.59	0.37	–
	O-05-01	50	1.59	0.34	0.58	0.42	5
	O-04-01	22	1.49	0.35	0.46	0.27	1
	O-03-01	8	1.53	0.32	0.49	0.41	–
	O-02-03	26	1.56	0.39	0.54	0.29	2
	O-02-02	18	1.52	0.35	0.51	0.34	–
	O-02-01	24	1.55	0.40	0.55	0.29	–
	O-01-01	35	1.57	0.33	0.57	0.44	1
Hiwassee	H-11-01	29	1.69	0.51	0.68	0.27	4
	H-09-03	20	1.74	0.66	0.72	0.11	2
	H-09-01	14	1.72	0.60	0.69	0.17	–
	H-09-02	16	1.71	0.71	0.68	-0.01	–
	H-08-07	6	1.70	0.57	0.65	0.21	–
	H-08-06	9	1.69	0.50	0.65	0.29	–
	H-08-04	15	1.69	0.54	0.67	0.22	–
	H-08-03	4	1.61	0.50	0.54	0.21	–
	H-07-03	58	1.73	0.59	0.72	0.20	2
	H-07-02	29	1.74	0.61	0.73	0.17	–
	H-07-01	4	1.63	0.42	0.55	0.37	4
	H-06-07	16	1.63	0.49	0.61	0.23	–
	H-06-05	50	4.19	0.53	0.60	0.13	4
	H-06-04	28	1.66	0.49	0.65	0.26	2
	H-06-02	33	1.70	0.57	0.69	0.19	2
	H-06-01	14	1.69	0.55	0.67	0.21	1
	H-12-06	11	4.22	0.54	0.60	0.16	1
	H-12-04	11	4.21	0.50	0.60	0.22	–
	H-05-01	50	1.70	0.63	0.70	0.10	1
	H-04-05	31	1.66	0.47	0.65	0.29	1
	H-04-04	44	1.74	0.40	0.74	0.46	11
	H-03-01	15	1.56	0.38	0.53	0.33	–
	H-02-01	25	1.52	0.41	0.51	0.23	–
	H-01-06	21	1.62	0.46	0.61	0.27	–
	H-01-02	29	1.56	0.49	0.55	0.12	1


*A_R_, allelic richness; H_O_, observed heterozygosity; H_E_, expected heterozygosity; F_IS_, inbreeding coefficient relative to the subpopulation.*

Forty-five private alleles were found in 4 Ocoee River and 13 Hiwassee River sampling sites, of which H-04-04 (11), O-05-01 (5), H-06-05 (4), H-07-01 (4), and H-11-01 (4) had the most, 5 locations (O-02-03, H-09-03, H-07-03, H-06-04, H-06-02) had 2 private alleles, and 7 locations had 1 private allele (**Table 1**). Private alleles were found at 12 loci, with PR002 (7) and PR031 (7) having the most and PR003 and PR035 having 1 each (**Table 1**). Thirty-four private alleles occurred at a frequency of <0.05, 5 at a frequency between 0.05 and 0.09, 5 at a frequency between 0.10 and 0.20, and 1 at a frequency greater than 0.25 (PR029, allele 245).

The variance components of the AMOVA analyses were highly significant at all hierarchical levels (*P* < 0.001, **Table 2**). Grouping of all sampling sites together indicated that most (68%) of the variation is explained within individuals, and 14 and 18% of the variation is due to differences among individuals within sampling sites and among sampling sites. Additional AMOVA grouping of sampling sites by river found similar results to the grouping of all sampling sites, with a greater percentage of the variation explained within individuals rather than among the individuals within the sampling site. Nearly 20% greater variation was found within individuals on the Hiwassee River as compared to the Ocoee River.

**Table 2:** Analysis of molecular variance (AMOVA) from nuclear microsatellite data collected from *Pityopsis ruthii* using Arlequin (version 3.5.1.2).

	*d.f.*	Sum of squares	Variance component	% of variation	*P*-value
**(A) Variance partition**					
Among populations	32	1086.36	0.63	17.90	<0.001
Among individuals within populations	781	2638.26	0.50	14.41	<0.001
Within individuals	814	1928.50	2.37	67.69	<0.001
Total	1627	5653.12	3.50	100	
Fixation indices: *F*_IS_ = 0.18, *F*_ST_ = 0.18, *F*_IT_ = 0.32					
**(B) Variance partition**					
Among populations	24	548.58	0.42	12.18	<0.001
Among individuals within populations	557	1937.06	0.43	12.29	<0.001
Within individuals	582	1527.00	2.62	75.53	<0.001
Total	1163	4012.64	3.47	100	
Fixation indices: *F*_IS_ = 0.14, *F*_ST_ = 0.12, *F*_IT_ = 0.25					
**(C) Variance partition**					
Among populations	7	271.02	0.63	20.69	<0.001
Among individuals within populations	224	701.20	0.70	22.84	<0.001
Within individuals	232	401.50	1.73	56.47	<0.001
Total	463	1373.72	3.07	100	
Fixation indices: *F*_IS_ = 0.28, *F*_ST_ = 0.21, *F*_IT_ = 0.44					


*A, the first analysis included all sampling sites as one hierarchical group; B, the second hierarchical analysis included all sampling sites on the Hiwassee River; C, the final analysis included all sampling sites on the Ocoee River; F_ST_, variance among sampling sites relative to the total variance; F_IS_, inbreeding coefficient of individuals relative to the population; F_IT_, variance in the total population; F_CT_, variance among groups relative to the total variance; F_SC_, variance among sampling sites within groups.*

Sampling sites of *P. ruthii* demonstrated a high level of differentiation (**Supplementary Tables S2, S3**). Pairwise comparisons of *F*_ST_ measures were all significantly different from zero for the Hiwassee River except for comparisons between nine sampling sites (**Supplementary Table S2**, *P* < 0.05), and the comparison for the Ocoee River were all significantly different than zero (**Supplementary Table S3**, *P* < 0.05). The greatest differentiation for the Hiwassee River sampling sites was observed between H-03-01 and H-08-03 (*F*_ST_= 0.31) and the lowest differentiation between H-06-05 and H-07-03 (*F*_ST_= 0.02). For the 300 pairwise sampling site comparisons of *F*_ST_ values for the Hiwassee River, 77 were less than 0.10, 180 were between 0.10 and 0.19, and 43 were greater than 0.20. For the Ocoee River sampling sites the greatest differentiation was observed between O-01-01 and O-04-01 (*F*_ST_= 0.28) and O-03-01 and O-04-01 (*F*_ST_= 0.28) and the lowest between O-02-01 and O-02-02 (*F*_ST_= 0.05), with 21 out of 28 comparisons greater than 0.15. Along the Hiwassee River, gene flow was highest between H-09-01 and H-09-03 (40.64) and lowest between H-02-01 and H-08-03 (0.60). Within the Ocoee River sampling sites, gene flow was highest between O-02-01 and O-02-02 and lowest between O-03-01 and O-04-01 (**Supplementary Table S3**). Overall, the Hiwassee River sampling sites had lower *F*_ST_ and much higher gene flow estimates than the Ocoee River sampling sites.

### Genetic Diversity in Chloroplast Microsatellites

We detected a total of 79 alleles among the seven chloroplast loci, with an average of 11.3 alleles per locus, ranging from a minimum of 5 to a maximum of 17 (**Supplementary Table S4**). Loci cpPR002 and cpPR010 showed the highest diversity (*h* = 0.54) whereas cpPR004 (*h* = 0.38) showed the least. Private alleles were identified at every locus, with a total of 15 across all loci and locations (**Table 3**). Thirteen sampling sites had private alleles: four from the Ocoee River and eleven from the Hiwassee River. In site H-04-04 private alleles were detected at multiple loci, whereas the other sampling sites had a single private allele. Site H-04-04 had the highest number of private alleles in both the nuclear and chloroplast data sets. Private alleles were detected at six other sampling sites for both nuclear and chloroplast loci: O-05-01, O-04-01, O-01-01, H-06-07, H-05-01, and H-04-05. Shannon’s Information Index was highest for Ocoee River site O-02-01 (1.07) and lowest for O-02-03 (0.62), whereas for the Hiwassee River sampling sites it was highest for H-01-06 (1.21) and lowest for H-08-07 (0.28). Diversity and unbiased diversity among the Ocoee River sampling sites were greatest in O-03-01 and lowest in O-04-01, and highest in H-01-06 and lowest in H-12-06 among the Hiwassee River sampling sites.

**Table 3:** Genetic variability of 7 chloroplast microsatellite loci estimated for 33 sampling sites of *Pityopsis ruthii*.

River	Sampling site	Sample size	N_A_	N_E_	I	h	uh	Private alleles
Ocoee	O-06-01	46	4.00	2.42	1.02	0.57	0.59	–
	O-05-01	50	3.71	2.05	0.84	0.46	0.47	1
	O-04-01	31	3.14	1.73	0.62	0.32	0.33	1
	O-03-01	8	3.29	2.75	1.05	0.61	0.71	–
	O-02-03	25	2.57	1.79	0.62	0.37	0.38	–
	O-02-02	17	3.43	2.48	0.98	0.55	0.59	–
	O-02-01	25	4.00	2.53	1.07	0.58	0.61	1
	O-01-01	33	3.57	1.84	0.79	0.44	0.45	1
Hiwassee	H-11-01	30	3.00	2.19	0.81	0.48	0.50	–
	H-09-03	19	3.29	2.15	0.86	0.48	0.51	–
	H-09-01	16	3.00	1.94	0.76	0.44	0.51	–
	H-09-02	14	3.71	2.98	1.10	0.60	0.65	1
	H-08-07	4	1.43	1.37	0.28	0.20	0.26	–
	H-08-06	8	2.14	1.46	0.47	0.27	0.32	–
	H-08-04	16	2.43	1.57	0.51	0.29	0.32	–
	H-08-03	4	2.00	1.70	0.56	0.37	0.50	–
	H-07-03	57	5.00	2.77	1.19	0.62	0.64	–
	H-07-02	29	3.29	2.36	0.88	0.49	0.52	1
	H-07-01	3	1.57	1.46	0.36	0.25	0.38	–
	H-06-07	17	2.86	2.02	0.76	0.44	0.49	–
	H-06-05	49	2.86	2.04	0.70	0.40	0.41	1
	H-06-04	28	3.71	2.69	1.09	0.61	0.63	–
	H-06-02	33	3.00	2.30	0.81	0.48	0.49	–
	H-06-01	15	3.29	2.35	0.98	0.57	0.62	–
	H-12-06	11	1.71	1.44	0.30	0.17	0.18	1
	H-12-04	11	2.71	1.97	0.72	0.42	0.47	1
	H-05-01	50	2.71	1.87	0.70	0.41	0.42	1
	H-04-05	31	2.86	1.69	0.58	0.32	0.33	1
	H-04-04	44	5.86	3.19	1.34	0.65	0.68	3
	H-03-01	15	3.71	2.39	0.98	0.52	0.59	–
	H-02-01	25	3.86	2.72	1.06	0.59	0.62	1
	H-01-06	20	4.29	2.95	1.21	0.64	0.69	–
	H-01-02	30	4.43	2.34	1.02	0.55	0.57	–


*N_A,_ Allele frequency; N_E,_ number of effective alleles; I, Shannon’s information index; h, diversity; uh, unbiased diversity.*

The haplotype analysis in ARLEQUIN detected 102 unique haplotypes within the 237 individuals from the Ocoee River sampling sites and 176 unique haplotypes within the 582 individuals from the Hiwassee River sampling sites, with only 5 complete haplotypes shared between the two rivers. The diversity within the two rivers was comparable, with the Ocoee River (*h* = 0.49) showing slightly higher diversity per sampling site than the Hiwassee River (*h* = 0.45) (**Table 3**).

A hierarchical AMOVA with two groups (**Table 4**), Hiwassee and Ocoee Rivers, revealed that differences between river only explained 5% of variation, whereas differences among sampling sites within groups explained 32% of variation and differences within sampling sites explained 63% of variation (*P* = 0.03). Among the Hiwassee River sampling sites, 37% of all variation could be explained among sampling sites, whereas differences within sampling sites explained 63% of variation (*P* < 0.01). Analysis of the Ocoee River populations revealed 22% variation among sampling sites and 78% variation explained by differences within sampling sites (*P* < 0.01). The Hiwassee River sampling sites had a higher *F*_ST_ (0.37) than the Ocoee River sampling sites (0.22).

**Table 4:** Analysis of molecular variance (AMOVA) from chloroplast microsatellite data collected from *Pityopsis ruthii* using Arlequin (version 3.5.1.2).

	*d.f*.	Sum of squares	Variance component	% of variation	*P*-value
**(A) Variance partition**					
Among groups	1	45.89	0.080 Va	4.66	<0.01
Among populations within groups	31	433.23	0.542 Vb	32.01	<0.01
Within populations	771	827.30	1.073 Vc	63.33	0.03
Total	803	1306.41	1.694	100	
Fixation indices: *F*_SC_ = 0.33, *F*_ST_ = 0.37, *F*_CT_ = 0.05					
**(B) Variance partition**					
Among populations	23	362.00	0.632 Va	37.12	<0.01
Within populations	542	579.93	1.070 Vb	62.88	<0.01
Total	565	941.94	1.702	100	
Fixation indices: *F*_ST_ = 0.37					
					
**(C) Variance partition**					
Among populations	7	67.34	0.299 Va	21.56	<0.01
Within populations	227	246.70	1.087 Vb	78.44	<0.01
Total	234	314.04	1.386	100	
Fixation indices: *F*_ST_ = 0.22					


*A, the first analysis included all sampling sites as one hierarchical group; B, the second hierarchical analysis included all sampling sites on the Hiwassee River; C, the final analysis included all sampling sites on the Ocoee River; F_ST_, variance among sampling sites relative to the total variance; F_IS_, inbreeding coefficient of individuals relative to the population; F_IT_, variance in the total population; F_CT_, variance among groups relative to the total variance; F_SC_, variance among sampling sites within groups.*

### Population Structure

When data for the Hiwassee and Ocoee Rivers were combined, STRUCTURE analysis of nuclear microsatellites found evidence for two distinct clusters, which are partitioned based on the two rivers that were sampled (**Supplementary Figure S1**). The STRUCTURE results using the chloroplast microsatellites differ from the nuclear microsatellites when all populations are combined. Admixture is more apparent in the chloroplast data than in the nuclear data as Δ*k* = 6 (**Supplementary Figure S1**). Clusters one and two include only sampling sites from the Hiwassee River and exhibit very little admixture, whereas the remaining four clusters exhibit a great deal of admixture and are difficult to distinguish. Cluster one (green) is composed of H-07-03, H-07-02, H-06-05, and H-12-06. Cluster two (red) is composed of H-11-01, H-06-01, and portions of H-06-02 and H-06-04.

When the chloroplast data was analyzed by river, cluster three in the analysis of Hiwassee shows strong similarities to cluster four in the combined chloroplast analysis; cluster four of the Hiwassee analysis and cluster one of the combined chloroplast analysis also show similarities. Sampling sites H-07-03 and H-04-04 show high admixture in both nuclear and chloroplast microsatellites.

**FIGURE 1:**
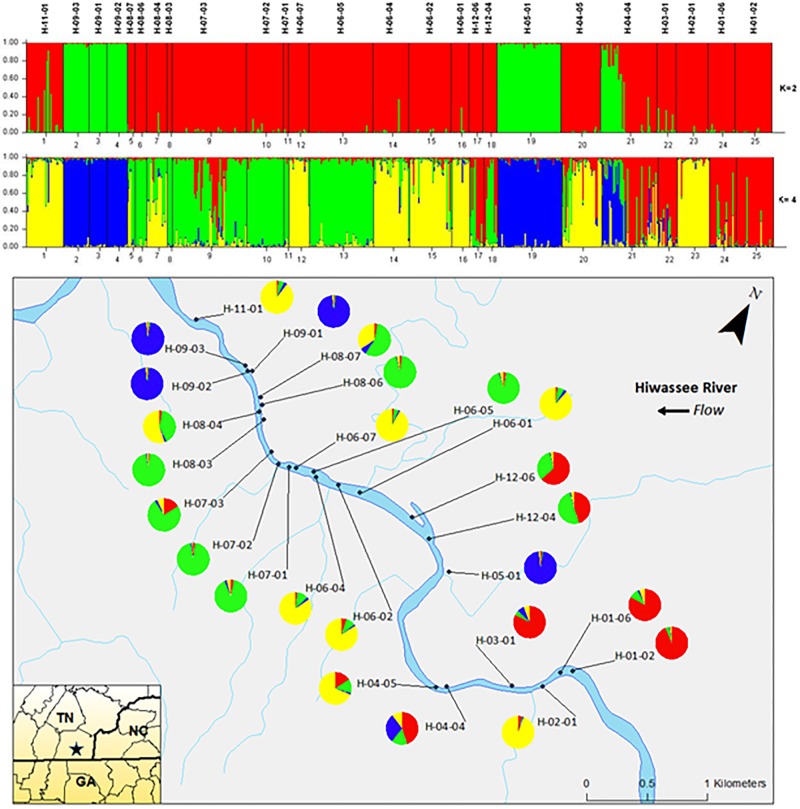
Bar plots of individual Bayesian assignment probabilities of nuclear microsatellites for *Pityopsis ruthii* Hiwassee River sampling sites using the program STRUCTURE for two or four clusters. Each vertical line represents an individual’s probability of belonging to one of k clusters (represented by different colors) or a combination if ancestry is mixed. Map of the sampled populations. Pie charts correspond to the population assignment for the four genetic groups defined by the Bayesian assignment of STRUCTURE.

**FIGURE 2:**
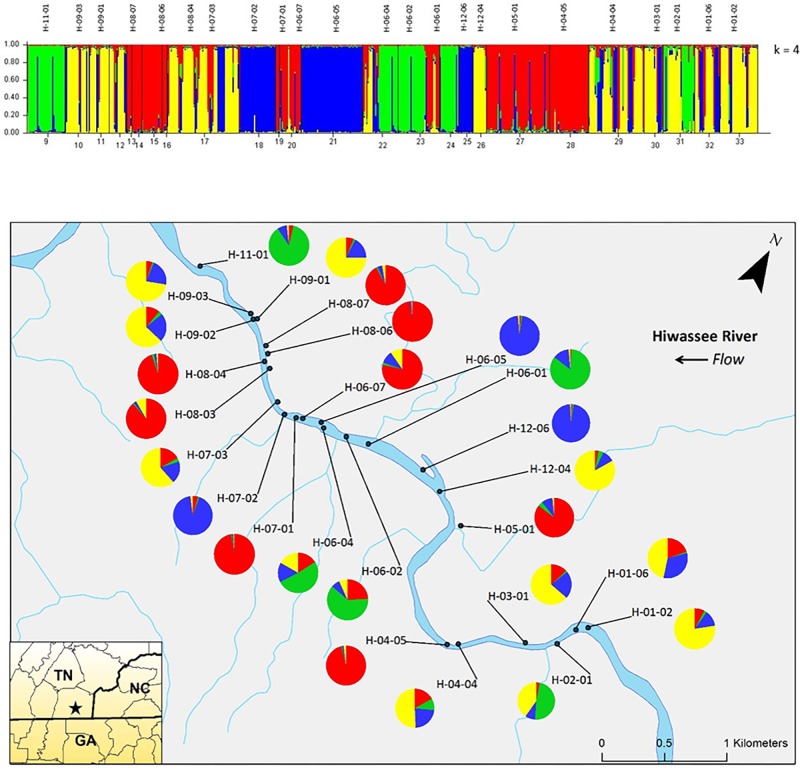
Bar plot of individual Bayesian probabilities of chloroplast microsatellites for *Pityopsis ruthii* Hiwassee River populations using the program STRUCTURE for four clusters. Each vertical line represents an individual’s probability of belonging to one of k clusters (represented by different colors) or a combination of if ancestry is mixed. Map of the sampled populations. Pie charts correspond to the population assignment for the four genetic groups defined by the Bayesian assignment of STRUCTURE.

Analyzing the data from each river separately revealed differences between the nuclear and chloroplast microsatellite data. Analysis of the nuclear microsatellites for the Hiwassee River sampling sites with STRUCTURE identified two genetically distinct clusters (**Figure 1**). Cluster one (green) is composed of individuals from H-09-03, H-09-02, H-09-01, H-05-01, and a portion of H-04-04, with the other cluster (red) composed of the remaining sampling sites. Analysis of the Hiwassee River sampling sites using chloroplast microsatellites revealed Δ*k* = 4 (**Figure 2**). Cluster one (green) is composed of H-11-01, H-06-04, H-06-02, and part of H-02-01. Cluster two (yellow) is composed of H-09-03, H-09-01, H-09-02, H-07-03, H-12-04, H-04-04, H-03-01, H-02-01, H-01-06, and H-01-02. Cluster three (red) includes H-08-07, H-08-06, H-08-04, H-08-03, H-06-07, H-05-01, and H-04-05. Cluster four (blue) is composed of H-07-02, H-06-05, and H-12-06, with very little admixture shown. Although admixture is evident among all sampling sites, it is higher within H-07-03, H-06-04, H-06-02, H-04-04, H-03-01, and H-02-01.

**FIGURE 3:**
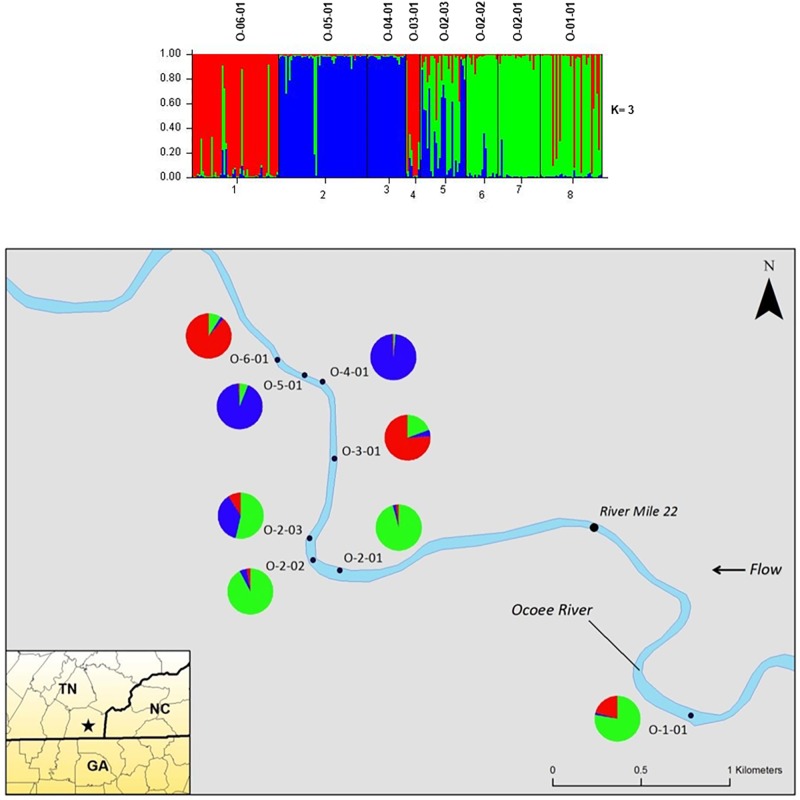
Bar plots of individual Bayesian assignment probabilities of nuclear microsatellites for *Pityopsis ruthii* Ocoee River populations using the program STRUCTURE for three clusters. Each vertical line represents an individual’s probability of belonging to one of *k* clusters (represented by different colors) or a combination of if ancestry is mixed. Map of the sampled populations. Pie charts correspond to the population assignment for the three genetic groups defined by the Bayesian assignment of STRUCTURE.

**FIGURE 4:**
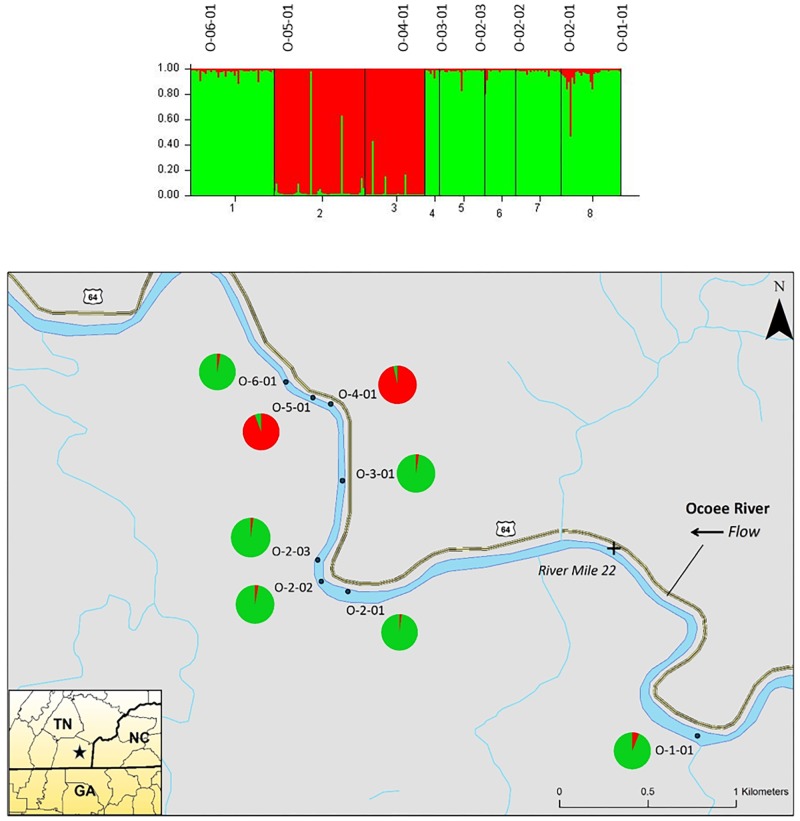
Bar plot of individual Bayesian assignment probabilities of chloroplast microsatellites for *Pityopsis ruthii* sampling sites along the Ocoee River using STRUCTURE for two clusters (*k* = 2). Each vertical line represents an individual’s probability of belonging to one of k clusters (represented by different colors) or a combination of if ancestry is mixed. Map of the sampled populations. Pie charts correspond to the population assignment for the two genetic groups defined by the Bayesian assignment of STRUCTURE.

The STRUCTURE analysis of nuclear microsatellites identified three clusters for the Ocoee River populations (**Figure 3**). Sampling sites O-06-01 and O-03-01 were in one cluster (red), O-05-01 and O-04-01 grouped in another cluster (blue), whereas O-02-03, O-02-02, O-02-01, and O-01-01 clustered into a third group (green). Although admixture is evident among all Ocoee River sampling sites, it is highest within O-06-01, O-03-01, O-02-03, and O-01-01. The STRUCTURE analysis with chloroplast microsatellites identified two clusters for the Ocoee sampling sites (**Figure 4**). Cluster one (green) includes O-06-01, O-03-01, O-02-03, O-02-02, O-02-01, and O-01-01. Cluster two (red) is composed of O-05-01 and O-04-01, which also clustered together in cluster one of the combined analysis. Admixture is low throughout both clusters, with only a few scattered individuals evidencing crossover between clusters.

Additionally, we randomly subsampled the sampling sites that had more than 25 individuals to ensure the accuracy of our STRUCTURE results with more uniform sampling size, as the program has been considered unreliable when uneven sampling occurs (Puechmaille, 2016). For the subsampled data set containing both rivers, we found Δ*k* = 3. When subsampled, Δ*k* = 5 for the Hiwassee River, whereas Δ*k* = 6 for the Ocoee River. The population structure differed between the subsampled results and previous analysis, detecting additional clusters. However, sampling sites clustered similarly between the subsampled and original data, with individuals in smaller populations showing higher levels of admixture but the main clusters remaining the same.

The pairwise correlation between geographic and genetic distance to determine isolation by distance using nuclear data shows a clear and significant (*P* < 0.01) separation the Hiwassee and Ocoee Rivers (**Supplementary Figure S2**). The separation is also noticeable and significant (*P* < 0.001) between rivers when using the chloroplast data from both rivers (**Supplementary Figure S3**). Isolation by distance is less apparent when the Hiwassee and Ocoee River populations are separated. For the Hiwassee River sampling sites, a significant relationship is apparent between geographic distance (km) and genetic distance in both the chloroplast (*P* < 0.001) and nuclear (*P* < 0.001) datasets (**Supplementary Figures S2, S3**). For the Ocoee River sampling sites, the Mantel test for the chloroplast microsatellites does not indicate a significant relationship between geographic and genetic distance, while the nuclear does (*P* < 0.001; **Supplementary Figures S2, S3**).

Wilcoxon tests to detect bottlenecks in *P. ruthii* sampling sites showed likely recent bottlenecks and loss of genetic diversity in all of the Hiwassee River clusters, as well as two of the Ocoee River sampling sites (**Supplementary Table S5**; *P* < 0.05). BOTTLENECK did not detect a bottleneck in the third cluster of the Ocoee River using any of the models.

## Discussion

*Pityopsis ruthii* is characterized by high levels of variation at nuclear microsatellite loci and moderate levels of variation for chloroplast microsatellite loci. Overall, *P. ruthii* has high levels of diversity compared to other species, both within Asteraceae and in other families. Expected heterozygosity is considerably higher (*H*_E_= 0.63) than that found in endemic cliff dwelling perennial species (*Opisthopappus longilobus*; *H*_E_= 0.20 and *O. taihangensis*; *H*_E_= 0.14) from China (Guo et al., 2013) and *Spiraea virginiana* (*H*_E_ = 0.39), a rare riparian shrub from the Southern Appalachian region of the United States (Brzyski and Culley, 2011). Within the Hiwassee River populations, slightly higher inbreeding coefficients (*F*_IS_) were found in some peripheral populations and suggest a higher degree of inbreeding in these populations compared to more central populations, though analyses of chloroplast microsatellites did not show a lack of diversity in those same populations. Diversity of *P. ruthii* (h = 0.46) estimated from chloroplast microsatellites was higher than seen in *O. longilobus* (*h* = 0.07) or *O. taihangensis* (*h* = 0.02) (Wang, 2013) and the Japanese riparian species *Ainsliaea faurieana* (*h* = 0.24), though the range within *A. faurieana* was comparable to that seen in *P. ruthii* (Mitsui et al., 2010). The Ocoee River sampling site mean diversity (*h* = 0.48) was higher than the Hiwassee River sampling site mean (0.45). When compared to similarly threatened species with narrow ranges (*Opisthopappus* spp. and *S. virginiana*), *P. ruthii* shows high levels of diversity for nuclear loci.

The chloroplast analyses revealed moderate levels of variation. We found greater genetic differentiation in chloroplast microsatellites than nuclear, as chloroplast microsatellites often show high *F*_ST_ values. The seven choloroplast loci had a higher number of alleles per locus and allelic frequency than loci used to study the widespread species *Chrysanthemum indicum* and *C. lavandulifolium* (Yang et al., 2006) and the endangered *O. longilobus* and *O. taihangensis* (Wang, 2013). Loci from *P. ruthii* also had an equivalent or greater number of alleles and allelic frequency than chloroplast loci used for *Begonia nelumbiifolia* and *B. heracleifolia*, which are also widespread plants (Twyford et al., 2013).

Regarding the proportion of diversity among rivers, the *F*_ST_ values for both rivers indicate high genetic differentiation among sampling sites. Our Mantel test results suggest that geography is highly correlated with genetic differentiation, as expected due to the clonal nature of *P. ruthii*, with individuals and sites showing greater differences when farther apart. Sampling sites with greater average pairwise differences have more genetically variable individuals than populations with lower average pairwise differences. Wright (1931) suggested when gene flow > 1, genetic differentiation among populations due to genetic drift can be prevented. Reduced gene flow can be expected to increase inbreeding within populations. However, in general, gene flow estimates greater than 0.5 indicate that migration is adequate to prevent genetic divergence of populations due to drift (Slatkin, 1987). All sampling sites along both rivers have gene flow estimates greater than 0.5 and are therefore not in immediate danger of genetic drift causing divergence among populations. Pairwise comparisons of gene flow estimates for sampling sites on the Ocoee River are in general much lower than those sampling sites on the Hiwassee River, as expected due to the much lower number of individuals.

We found significant (*P* > 0.001) *F*_IS_ values for all sampling sites regardless of river indicating an excess of homozygosity and inbreeding. Though significant, *F*_IS_ values for *P. ruthii* were comparatively low when compared to other species such as *A. faurieana* (Mitsui et al., 2010). Inbreeding in the case of *P. ruthii* could be attributed to mating among relatives, which could lead to lower seed viability or seedling vigor. Pollen and seed dispersal are the main mechanisms for natural gene flow. In order to evaluate the results of our study, we need to take into consideration what is known about pollen and seed dispersal of *P. ruthii*. Seed distribution is thought to be adapted for water dispersal or rolling around on the rock substrate until a seed is lodged into a suitable crevice or blow into the water by the wind (Clebsch and Sloan, 1993, Unpublished). Germination of seedlings in wild populations has been observed and the mortality was higher than 90% after 1 year (Clebsch and Sloan, 1993, Unpublished). Further studies are needed to determine if seedlings at *P. ruthii* sampling sites with high inbreeding coefficients are suffering from inbreeding depression or if seedling recruitment is limited.

Considering that this study captures the genetic variation from a single time point for these sampling sites, determining whether genetic variation is increasing, decreasing, or stable is difficult. Genetic diversity does not increase with any regularity within sampling sites downstream, at either the nuclear or chloroplast microsatellite loci. The diversity among chloroplast loci throughout the sampling sites suggests that *P. ruthii* seed travels downstream, whereas nuclear loci diversity includes pollen dispersal. Both nuclear and chloroplast data provide evidence that gene flow within *P. ruthii* is multidirectional along both the Ocoee and Hiwassee Rivers. In riparian systems, the unidirectional dispersal hypothesis indicates a hydrochoric system with seed dispersal solely based on water flow (Werth et al., 2014). As such, wind, pollinators, and other dispersal mechanisms do not facilitate high levels of gene flow upriver in systems with unidirectional gene flow. Though some systems provide evidence for the unidirectional dispersal hypothesis (e.g., Liu et al., 2006; Mitsui et al., 2010), several others do not (Honnay et al., 2010; Werth and Scheidegger, 2014). For *P. ruthii*, levels of diversity and gene flow along both the Ocoee and Hiwassee Rivers reflect a healthy exchange of material both upstream and downstream.

Recent bottlenecks were detected in multiple clusters of sampling sites, indicating a loss of genetic diversity, which could impact adaptation. Cluster three of the Ocoee River from the nuclear microsatellites (**Figure 3**; O-02-03, O-02-02, O-02-01, and O-01-01) showed no signs of a bottleneck, perhaps due to the inclusion of four sampling sites, two of which (O-01-01 and O-02-03) show high admixture with the nuclear data. Coupled with the higher levels of gene flow among these sampling sites and high admixture in the two sampling sites furthest upstream, the lack of bottlenecking may indicate a founder effect. The four sampling sites that make up cluster three are located upstream from the other sites along the Ocoee, allowing little gene flow from the downstream sampling sites, but also showing little gene flow from cluster three to other locations, resulting in bottlenecks downstream. Considering the geographical proximity to one another, the outcrossing breeding system, and habitat continuity, we should expect genetic exchange among populations through pollen or seed dispersal. However, asynchronous flowering within sampling sites could compound inbreeding and explain the high *F*_IS_ values observed. Variation of flowering times is not uncommon; flowering starts as early as late July to early August and continues until late October to early November. Mating of individuals from each group could lead to increased inbreeding, explaining the inbreeding coefficients within sampling sites.

Additionally, drought appears to be a factor in maintaining habitat for some sampling sites of *P. ruthii* (Moore et al., 2016). Site H-04-04 has the highest number of private alleles for both the nuclear and chloroplast loci. H-04-04 is considered a drought-maintained sampling site due to the lack of inundating flows, and other drought-tolerant species to compete for resources are currently lacking (Moore et al., 2016). This drought-tolerance at H-04-04 seems to allow diversification from other sites, leading to higher number of private alleles and low *F*_ST_ values. High admixture is apparent in this sampling site in both the nuclear and chloroplast datasets, which could indicate recruitment to this site from other populations, since conditions are more favorable for *P. ruthii*.

Fewer alleles and lower overall diversity was seen in chloroplast microsatellites when compared to nuclear microsatellites. This was expected due to the lack of recombination and smaller effective population size of chloroplast loci. The clear admixture between rivers in the STRUCTURE results from the chloroplast data is not apparent in the nuclear data. Coupled with the AMOVA results indicating a lack of variance between the Ocoee and Hiwassee River sampling sites, the differences between chloroplast and nuclear data are evident. Due to a lack of recombination, mutation rates in chloroplast genomes are lower than those in nuclear genomes; chloroplast markers allow a glimpse into a longer-term evolutionary scale than provided by nuclear markers alone. One possibility for the lack of allelic fixation in the chloroplast data is that the two populations of *P. ruthii* were once a single, widespread population that fragmented due to unknown causes, leading to higher levels of genetic drift in the genome and lower levels in the more conserved plastome. The shorter-term scale of nuclear data shows the effects of more recent fragmentation of the species, which is ultimately more informative for conservation than the chloroplast data.

Dams on the Hiwassee and Ocoee Rivers have altered the hydrology of both rivers where *P. ruthii* occur, though it is not clear what effect damming of the river and augmented flows have on seed recruitment. The lack of information on seed recruitment and habitat loss, coupled with high mortality of seedlings within natural populations pose challenges to developing strategies to protect sustainability of these populations. Another scenario is that the detected gene flow levels at least in part reflect natural gene flow. Lower levels of gene flow in some sampling sites could also be attributed to geographic isolation, which could restrict effective pollen or seed exchange among sampling sites.

Coupled with the molecular markers in this diversity study and the information now available on population structure, preventing further loss of diversity and protecting *Pityopsis ruthii* is becoming reality. Molecular markers are commonly used in maintenance and selection of germplasm collections, and chloroplast microsatellites in particular have been used to much advantage in several species (Balas et al., 2014). A core germplasm collection should include the majority of diversity without excessive redundancy, which we can now access from the frequency of private alleles detected using the microsatellites outlined in this study. Currently, the germplasm collection for *P. ruthii* is curated at the North Carolina Botanical Garden. The diversity maintained in this collection has not been characterized and it is now possible to assemble a core germplasm collection for *P. ruthii*. Including accessions which show high polymorphism among our nuclear and chloroplast loci will allow greater levels of diversity to be captured for future use.

Ongoing pollination studies and reintroduction efforts add to the effort base of knowledge on the ecology of this endangered species (Wadl et al., 2014, 2018; Moore et al., 2016). With the habitat and topography of *P. ruthii*, surrounded by high ridges and ineffective seed dispersal mechanisms, the species may not be able to migrate with warming climates. Additionally, the rivers show differing levels of growth, with sampling sites along the Ocoee River exhibiting greater numbers of flowers per plant and a lower level of competition with other herbaceous and woody plants (Moore et al., 2016). Moore et al. (2016) hypothesize that cyclical drought also plays a role in maintaining the rocky habitat necessary to support subpopulations of *P. ruthii*. Along with understanding the ecology of the species, a viable method of introducing diversity into the natural habitat is necessary. Cultivation of plants is possible through both stem cuttings and tissue culture, providing methods for reintroduction studies; it is feasible to grow *P. ruthii* seedlings *in vitro* and transplant seamlessly into the natural habitat (Wadl et al., 2014, 2018).

We reject our null hypothesis regarding population structuring and accept the alternative hypothesis that each river includes multiple viable breeding populations which are distinct from one another. Currently, each discrete site is managed as a separate population, though our study shows no evidence for such fragmentation. Rather, we posit that multiple sampling sites have similar genetics and therefore can be managed as one larger population. This is especially evident when viewing data from the nuclear microsatellite markers. Managing the species using a framework with two to four populations along the Hiwassee River and two to three along the Ocoee River will allow researchers to use plants in larger placement areas for augmentation, reintroduction, and/or translocation studies to add diversity to a particular population. Our work in identifying conservation units for this species and understanding the underlying genetics and ecology of the species will inform conservation practices in the future, as well as further study into the entire genus *Pityopsis*, and has provided a relatively easy and cost-effective way to follow genetic diversity in existing, augmented, or reintroduced populations over time.

## Author Contributions

AD, ES, MS, RT, and PW conceived and designed the experiments. AD, EH, and PW performed the experiments. AD, EH, ÐH, TR, and PW analyzed the data. RT, TR, MS, and PW contributed reagents, materials, and analysis tools. EH, PW, and AD wrote the paper. EH, AD, ÐH, TR, ES, MS, RT, and PW read and approved the manuscript.

## Conflict of Interest Statement

The authors declare that the research was conducted in the absence of any commercial or financial relationships that could be construed as a potential conflict of interest.

## Supplementary Material

FIGURE S1 Bar plot **(top)** of individual Bayesian assignment probabilities for nuclear microsatellites for the Hiwassee and Ocoee Rivers sampling sites of *Pityopsis ruthii* using the program STRUCTURE for two clusters (*k* = 2). Bar plot **(lower)** of individual Bayesian assignment probabilities for chloroplast microsatellites for the Hiwassee and Ocoee Rivers sampling sites using the program STRUCTURE for six clusters (*k* = 6). The *x*-axis indicates the individuals sampled and the *y*-axis indicates the assignment probability of individuals to each of the two clusters. Each vertical line represents an individual’s probability of belonging to one of *k* clusters (represented by different colors) or a combination of if ancestry is mixed.

FIGURE S2 Isolation by distance of *Pityopsis ruthii* populations using nuclear microsatellites. **(A)** Correlation between pairwise genetic distance values and geographic distance for all populations (*P* < 0.01). **(B)** Correlation between pairwise genetic distance values and geographic distance for Hiwassee River populations (*P* < 0.001). **(C)** Correlation between pairwise genetic distance values and geographic distance for Ocoee River populations (*P* < 0.001).

FIGURE S3 Isolation by distance of *Pityopsis ruthii* populations using chloroplast microsatellites. **(A)** Correlation between pairwise genetic distance values and geographic distance for all locations (*P* < 0.001); **(B)** Correlation between pairwise genetic distance values and geographic distance for locations along the Hiwassee River (*P* < 0.001); **(C)** Correlation between pairwise genetic distance and geographic distance for locations along the Ocoee River (*P* > 0.05).

TABLE S1 Summary statistics for 12 nuclear microsatellite loci in *Pityopsis ruthii* populations.

TABLE S2 Pairwise *F*_ST_ values (below diagonal) and gene flow estimates (above diagonal) for *Pityopsis ruthii* sampling sites on the Hiwassee River.

TABLE S3 Pairwise *F*_ST_ values (below diagonal) and gene flow estimates (above diagonal) for *Pityopsis ruthii* sampling sites on the Ocoee River.

TABLE S4 Characterization of seven chloroplast microsatellite markers from *Pityopsis ruthii*.

TABLE S5 Wilcoxon tests for recent bottlenecks using the program BOTTLENECK for *Pityopsis ruthii* sampling sites. The *P*-values are reported for the infinite allele model (IAM), two-phase mutational model (TPM), and stepwise mutational model (SMM).

DATA SHEET S1 Raw and binned microsatellite allelic data for all individuals used in analyses.
