# Supplementary figures and images for: Population Structure and Genetic Diversity Within the Endangered Species Pityopsis ruthii (Asteraceae)
Source: Front Plant Sci. 2018 Jul 11;9:943. doi: 10.3389/fpls.2018.00943 (PMC6050971; doi:10.3389/fpls.2018.00943)

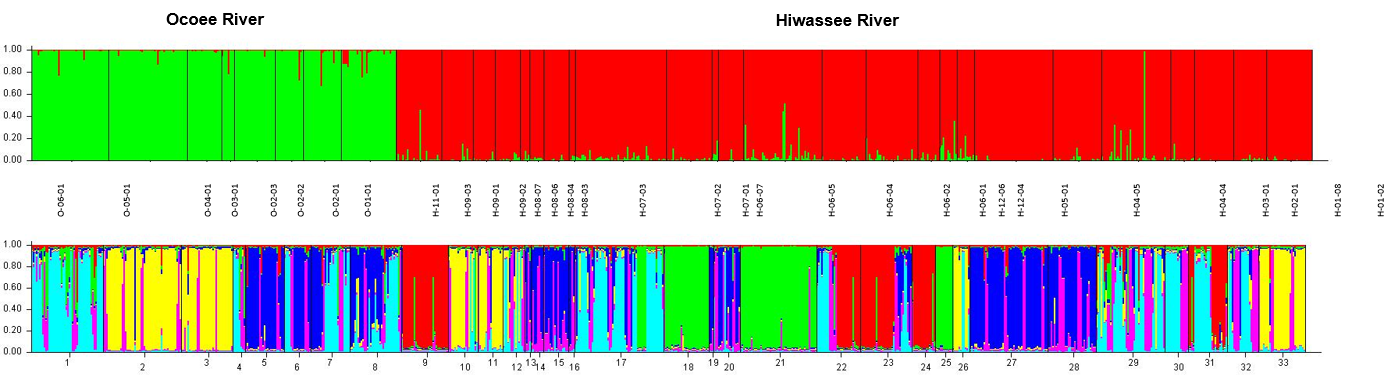

Supplement: FIGURE S1 — Bar plot (top) of individual Bayesian assignment probabilities for nuclear microsatellites for the Hiwassee and Ocoee Rivers sampling sites of Pityopsis ruthii using the program STRUCTURE for two clusters (k = 2). Bar plot (lower) of individual Bayesian assignment probabilities for chloroplast microsatellites for the Hiwassee and Ocoee Rivers sampling sites using the program STRUCTURE for six clusters (k = 6). The x-axis indicates the individuals sampled and the y-axis indicates the assignment probability of individuals to each of the two clusters. Each vertical line represents an individual’s probability of belonging to one of k clusters (represented by different colors) or a combination of if ancestry is mixed. [file Image_1.TIF]

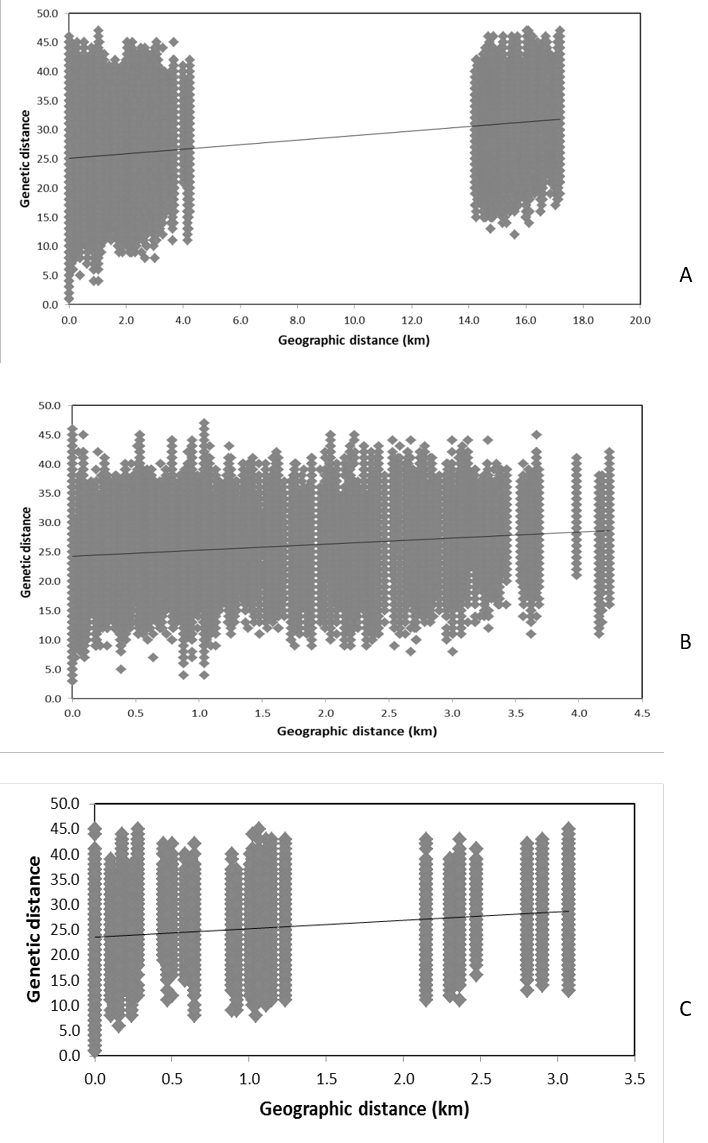

Supplement: FIGURE S2 — Isolation by distance of Pityopsis ruthii populations using nuclear microsatellites. (A) Correlation between pairwise genetic distance values and geographic distance for all populations (P < 0.01). (B) Correlation between pairwise genetic distance values and geographic distance for Hiwassee River populations (P < 0.001). (C) Correlation between pairwise genetic distance values and geographic distance for Ocoee River populations (P < 0.001). [file Image_2.TIF]

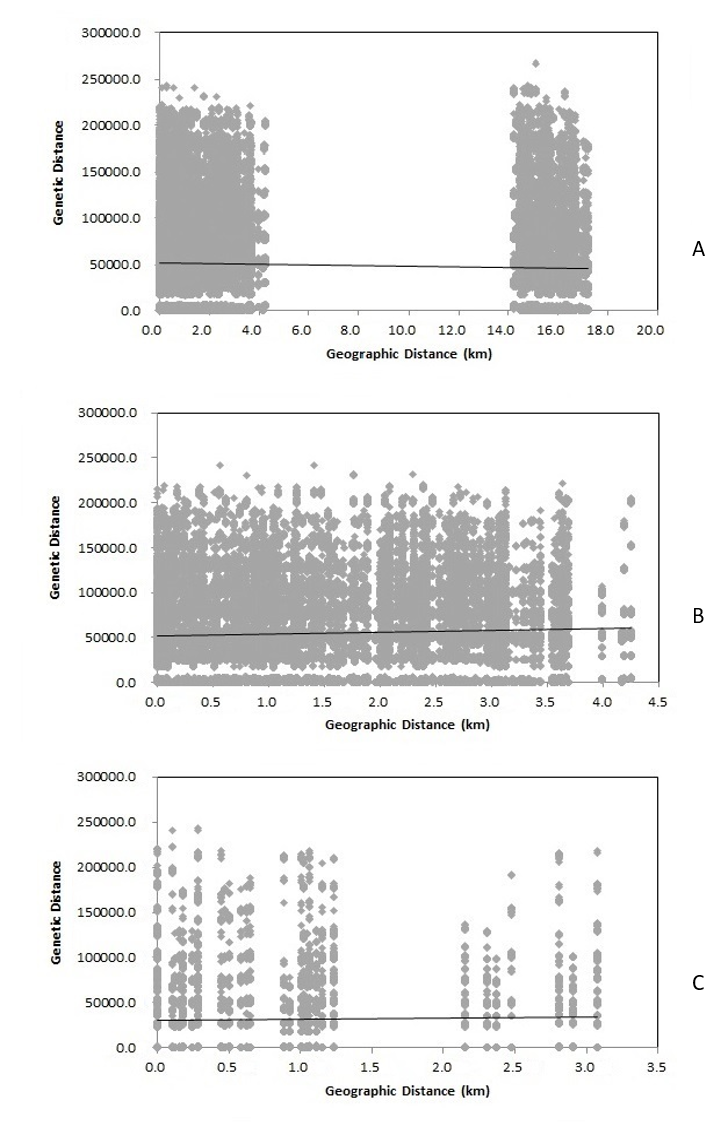

Supplement: FIGURE S3 — Isolation by distance of Pityopsis ruthii populations using chloroplast microsatellites. (A) Correlation between pairwise genetic distance values and geographic distance for all locations (P < 0.001); (B) Correlation between pairwise genetic distance values and geographic distance for locations along the Hiwassee River (P < 0.001); (C) Correlation between pairwise genetic distance and geographic distance for locations along the Ocoee River (P > 0.05). [file Image_3.TIF]
