# Supplementary material for: Population Structure and Genetic Diversity Within the Endangered Species Pityopsis ruthii (Asteraceae)
Source: Front Plant Sci. 2018 Jul 11;9:943. doi: 10.3389/fpls.2018.00943 (PMC6050971; doi:10.3389/fpls.2018.00943)
Supplement: TABLE S1 — Summary statistics for 12 nuclear microsatellite loci in Pityopsis ruthii populations. [file Table_1.DOCX]

| **Table S1.** Summary statistics for 12 nuclear microsatellite loci in *Pityopsis ruthii* populations. | | | | | | | | | |
| --- | --- | --- | --- | --- | --- | --- | --- | --- | --- |
| Locus | Repeat motif | *A* | *A*_R_ | *H*_O_ | *H*_E_ | *F*_IS_ | *F*_IT_ | *F*_ST_ | *N*_m_ |
| PR002 | (TG)_9_ | 15 | 1.68 | 0.50 | 0.52 | 0.02 | 0.25 | 0.24 | 0.78 |
| PR003 | (TG)_14_ | 9 | 1.78 | 0.42 | 0.59 | 0.26 | 0.46 | 0.27 | 0.69 |
| PR005 | (CT)_7_ | 16 | 1.85 | 0.76 | 0.63 | -0.23 | 0.11 | 0.27 | 0.66 |
| PR006 | (CA)_16_ | 17 | 1.81 | 0.33 | 0.67 | 0.48 | 0.59 | 0.21 | 0.94 |
| PR009 | (GT)_11_ | 16 | 1.80 | 0.43 | 0.62 | 0.27 | 0.53 | 0.36 | 0.45 |
| PR020 | (GT)_8_ | 13 | 1.79 | 0.48 | 0.68 | 0.26 | 0.39 | 0.18 | 1.16 |
| PR027 | (GTGTC)_5_ | 18 | 1.78 | 0.50 | 0.62 | 0.17 | 0.35 | 0.23 | 0.86 |
| PR028 | (GT)_10_ | 9 | 1.69 | 0.28 | 0.54 | 0.45 | 0.59 | 0.26 | 0.70 |
| PR029 | (GT)_3_A(GT)_8_ | 24 | 1.89 | 0.66 | 0.77 | 0.10 | 0.26 | 0.17 | 1.24 |
| PR030 | (AC)_12_ | 21 | 1.89 | 0.53 | 0.77 | 0.28 | 0.41 | 0.18 | 1.15 |
| PR031 | (GT)_9_AA(GT)_5_ | 19 | 1.82 | 0.25 | 0.57 | 0.54 | 0.70 | 0.35 | 0.47 |
| PR035 | (GT)_5_A(TG)_7_(AG)_15_ | 16 | 1.84 | 0.71 | 0.76 | 0.04 | 0.16 | 0.12 | 1.76 |
| Mean |  | 16 | 1.80 | 0.49 | 0.65 | 0.22 | 0.40 | 0.24 | 0.90 |
| *A=* number of alleles, *A*_R_= allelic richness, *H*_O_= observed heterozygosity, *H*_E_= expected heterozygosity, *F*_IS_= inbreeding coefficient relative to the subpopulation, *F*_IT_= inbreeding coefficient relative to total number of individuals, *F*_ST_= fixation index, *N*_m_= estimated gene flow | | | | | | | | | |
