# Supplementary material for: Population Structure and Genetic Diversity Within the Endangered Species Pityopsis ruthii (Asteraceae)
Source: Front Plant Sci. 2018 Jul 11;9:943. doi: 10.3389/fpls.2018.00943 (PMC6050971; doi:10.3389/fpls.2018.00943)
Supplement: TABLE S2 — Pairwise FST values (below diagonal) and gene flow estimates (above diagonal) for Pityopsis ruthii sampling sites on the Hiwassee River. [file Table_2.DOCX]

| **Table S2.** Pairwise F_ST_ values (below diagonal) and gene flow estimates (above diagonal) for *Pityopsis* *ruthii* subpopulations on the Hiwassee River. | | | | | | | | | | | | | | | | | | | | | | | | | |
| --- | --- | --- | --- | --- | --- | --- | --- | --- | --- | --- | --- | --- | --- | --- | --- | --- | --- | --- | --- | --- | --- | --- | --- | --- | --- |
|  | H-11-01 | H-09-03 | H-09-01 | H-09-02 | H-08-07 | H-08-06 | H-08-04 | H-08-03 | H-07-03 | H-07-02 | H-07-01 | H-06-07 | H-06-05 | H-06-04 | H-06-02 | H-06-01 | H-12-06 | H-12-04 | H-05-01 | H-04-05 | H-04-04 | H-03-01 | H-02-01 | H-01-06 | H-01-02 |
| H-11-01 | 0.00 | 1.53 | 1.70 | 1.36 | 3.11 | 1.68 | 3.21 | 1.71 | 2.67 | 1.54 | 1.77 | 1.72 | 3.12 | 1.83 | 2.21 | 2.07 | 1.63 | 1.68 | 1.17 | 1.90 | 1.59 | 1.27 | 1.34 | 1.05 | 0.81 |
| H-09-03 | 0.14 | 0.00 | 40.64 | 5.84 | 1.68 | 1.19 | 1.32 | 0.96 | 1.68 | 0.97 | 1.30 | 1.08 | 1.36 | 1.27 | 1.28 | 1.38 | 1.43 | 1.34 | 3.13 | 1.30 | 2.01 | 1.65 | 0.73 | 1.24 | 0.82 |
| H-09-01 | 0.13 | 0.01^ns^ | 0.00 | 22.61 | 1.83 | 1.30 | 1.38 | 0.98 | 1.72 | 1.04 | 1.45 | 1.05 | 1.42 | 1.36 | 1.44 | 1.49 | 1.66 | 1.55 | 2.85 | 1.46 | 2.20 | 1.64 | 0.77 | 1.16 | 0.74 |
| H-09-02 | 0.16 | 0.04 | 0.01^ns^ | 0.00 | 1.54 | 1.10 | 1.09 | 0.95 | 1.45 | 0.99 | 1.19 | 0.87 | 1.12 | 1.15 | 1.19 | 1.17 | 1.24 | 1.20 | 2.74 | 1.03 | 1.54 | 1.17 | 0.64 | 0.96 | 0.66 |
| H-08-07 | 0.07 | 0.13 | 0.12 | 0.14 | 0.00 | 4.37 | 8.43 | 4.41 | 2.98 | 2.51 | 2.35 | 1.22 | 3.00 | 1.45 | 1.63 | 1.87 | 2.57 | 1.82 | 1.51 | 1.43 | 1.84 | 1.02 | 0.77 | 1.32 | 0.77 |
| H-08-06 | 0.13 | 0.17 | 0.16 | 0.19 | 0.05^ns^ | 0.00 | 2.96 | 3.78 | 3.63 | 1.64 | 2.55 | 1.10 | 3.98 | 1.57 | 1.68 | 1.59 | 4.43 | 2.22 | 0.96 | 1.19 | 1.82 | 0.86 | 0.70 | 1.40 | 1.05 |
| H-08-04 | 0.07 | 0.16 | 0.15 | 0.19 | 0.03^ns^ | 0.08 | 0.00 | 4.04 | 3.09 | 1.75 | 2.47 | 1.72 | 4.06 | 2.67 | 2.61 | 2.98 | 2.23 | 1.65 | 1.13 | 1.66 | 1.81 | 1.04 | 0.94 | 1.16 | 0.82 |
| H-08-03 | 0.13 | 0.21 | 0.20 | 0.21 | 0.05^ns^ | 0.06^ns^ | 0.06^ns^ | 0.00 | 3.08 | 1.73 | 2.24 | 0.95 | 6.53 | 1.37 | 1.35 | 1.24 | 2.06 | 1.51 | 0.98 | 1.02 | 1.37 | 0.57 | 0.60 | 0.90 | 0.71 |
| H-07-03 | 0.09 | 0.13 | 0.13 | 0.14 | 0.08 | 0.07 | 0.08 | 0.08 | 0.00 | 3.00 | 4.30 | 2.21 | 10.53 | 3.49 | 3.02 | 3.27 | 3.51 | 5.26 | 1.40 | 2.79 | 5.35 | 2.31 | 1.45 | 2.97 | 1.68 |
| H-07-02 | 0.14 | 0.21 | 0.19 | 0.20 | 0.09 | 0.13 | 0.12 | 0.13^ns^ | 0.08 | 0.00 | 2.64 | 1.47 | 2.94 | 1.55 | 1.81 | 1.69 | 1.74 | 1.25 | 1.15 | 1.68 | 1.82 | 0.65 | 0.88 | 1.04 | 0.74 |
| H-07-01 | 0.13 | 0.16 | 0.15 | 0.17 | 0.10 | 0.09 | 0.09 | 0.10 | 0.06 | 0.09 | 0.00 | 1.97 | 3.56 | 2.06 | 2.39 | 1.96 | 2.88 | 2.56 | 1.30 | 1.91 | 2.64 | 1.33 | 1.05 | 1.29 | 1.10 |
| H-06-07 | 0.13 | 0.19 | 0.19 | 0.22 | 0.17 | 0.19 | 0.13 | 0.21 | 0.10 | 0.15 | 0.11 | 0.00 | 2.34 | 2.66 | 4.15 | 3.44 | 1.27 | 1.56 | 1.21 | 3.20 | 2.92 | 1.17 | 1.67 | 1.00 | 0.91 |
| H-06-05 | 0.07 | 0.16 | 0.15 | 0.18 | 0.08 | 0.06 | 0.06 | 0.04^ns^ | 0.02 | 0.08 | 0.07 | 0.10 | 0.00 | 4.22 | 4.80 | 4.76 | 1.74 | 2.16 | 1.21 | 2.94 | 5.18 | 1.83 | 1.37 | 2.19 | 1.24 |
| H-06-04 | 0.12 | 0.16 | 0.16 | 0.18 | 0.15 | 0.14 | 0.09 | 0.15 | 0.07 | 0.14 | 0.11 | 0.09 | 0.06 | 0.00 | 3.59 | 4.25 | 1.86 | 1.65 | 1.05 | 1.92 | 2.19 | 1.27 | 1.13 | 1.13 | 0.85 |
| H-06-02 | 0.10 | 0.16 | 0.15 | 0.17 | 0.13 | 0.13 | 0.09 | 0.16 | 0.08 | 0.12 | 0.10 | 0.06 | 0.05 | 0.07 | 0.00 | 9.56 | 2.34 | 2.79 | 1.32 | 3.55 | 3.40 | 1.42 | 1.61 | 1.35 | 1.10 |
| H-06-01 | 0.11 | 0.15 | 0.14 | 0.18 | 0.12 | 0.14 | 0.08 | 0.17 | 0.07 | 0.13 | 0.11 | 0.07 | 0.05 | 0.06 | 0.03 | 0.00 | 2.34 | 2.53 | 1.27 | 3.40 | 3.14 | 1.32 | 1.55 | 1.35 | 0.88 |
| H-12-06 | 0.13 | 0.15 | 0.13 | 0.17 | 0.09 | 0.05 | 0.10 | 0.11 | 0.07 | 0.13 | 0.08 | 0.16 | 0.13 | 0.12 | 0.10 | 0.10 | 0.00 | 2.17 | 1.32 | 1.88 | 4.31 | 1.40 | 0.88 | 2.53 | 1.99 |
| H-12-04 | 0.13 | 0.16 | 0.14 | 0.17 | 0.12 | 0.10 | 0.13 | 0.14 | 0.05 | 0.17 | 0.09 | 0.14 | 0.10 | 0.13 | 0.08 | 0.09 | 0.10 | 0.00 | 1.37 | 2.78 | 5.62 | 1.41 | 1.40 | 6.04 | 2.21 |
| H-05-01 | 0.18 | 0.07 | 0.08 | 0.08 | 0.14 | 0.21 | 0.18 | 0.20 | 0.15 | 0.18 | 0.16 | 0.17 | 0.17 | 0.19 | 0.16 | 0.17 | 0.16 | 0.15 | 0.00 | 1.34 | 1.93 | 1.32 | 0.78 | 1.27 | 0.91 |
| H-04-05 | 0.12 | 0.16 | 0.15 | 0.20 | 0.15 | 0.17 | 0.13 | 0.20 | 0.08 | 0.13 | 0.12 | 0.07 | 0.08 | 0.12 | 0.07 | 0.07 | 0.12 | 0.08 | 0.16 | 0.00 | 3.72 | 1.79 | 2.86 | 1.94 | 1.29 |
| H-04-04 | 0.14 | 0.11 | 0.10 | 0.14 | 0.12 | 0.12 | 0.12 | 0.15 | 0.05 | 0.12 | 0.09 | 0.08 | 0.05 | 0.10 | 0.07 | 0.07 | 0.06 | 0.04 | 0.11 | 0.06 | 0.00 | 2.01 | 1.27 | 3.20 | 2.43 |
| H-03-01 | 0.16 | 0.13 | 0.13 | 0.18 | 0.20 | 0.22 | 0.19 | 0.31 | 0.10 | 0.28 | 0.16 | 0.18 | 0.12 | 0.16 | 0.15 | 0.16 | 0.15 | 0.15 | 0.16 | 0.12 | 0.11 | 0.00 | 0.73 | 1.49 | 1.01 |
| H-02-01 | 0.16 | 0.25 | 0.25 | 0.28 | 0.24 | 0.26 | 0.21 | 0.30 | 0.15 | 0.22 | 0.19 | 0.13 | 0.16 | 0.18 | 0.14 | 0.14 | 0.22 | 0.15 | 0.24 | 0.08 | 0.17 | 0.26 | 0.00 | 0.90 | 0.75 |
| H-01-06 | 0.19 | 0.17 | 0.18 | 0.21 | 0.16 | 0.15 | 0.18 | 0.22 | 0.08 | 0.19 | 0.16 | 0.20 | 0.10 | 0.18 | 0.16 | 0.16 | 0.09 | 0.04 | 0.16 | 0.11 | 0.07 | 0.14 | 0.22 | 0.00 | 3.54 |
| H-01-02 | 0.24 | 0.23 | 0.25 | 0.28 | 0.24 | 0.19 | 0.23 | 0.26 | 0.13 | 0.25 | 0.19 | 0.22 | 0.17 | 0.23 | 0.19 | 0.22 | 0.11 | 0.10 | 0.22 | 0.16 | 0.09 | 0.20 | 0.25 | 0.07 | 0.00 |
