# Supplementary material for: Population Structure and Genetic Diversity Within the Endangered Species Pityopsis ruthii (Asteraceae)
Source: Front Plant Sci. 2018 Jul 11;9:943. doi: 10.3389/fpls.2018.00943 (PMC6050971; doi:10.3389/fpls.2018.00943)
Supplement: TABLE S3 — Pairwise FST values (below diagonal) and gene flow estimates (above diagonal) for Pityopsis ruthii sampling sites on the Ocoee River. [file Table_3.DOCX]

| **Table S3.** Pairwise *F*_ST_ values (below diagonal) and gene flow estimates (above diagonal) for *Pityopsis ruthii* subpopulations on the Ocoee River. | | | | | | | | | |
| --- | --- | --- | --- | --- | --- | --- | --- | --- | --- |
|  | O-06-01 | O-05-01 | O-04-01 | O-03-01 | O-02-03 | O-02-02 | O-02-01 | O-01-01 |  |
| O-06-01 | 0.00 | 1.00 | 0.85 | 0.78 | 1.13 | 1.46 | 1.20 | 0.88 |  |
| O-05-01 | 0.20 | 0.00 | 2.48 | 0.82 | 1.74 | 1.35 | 1.08 | 0.82 |  |
| O-04-01 | 0.23 | 0.09 | 0.00 | 0.64 | 1.36 | 0.99 | 0.87 | 0.66 |  |
| O-03-01 | 0.24 | 0.23 | 0.28 | 0.00 | 1.10 | 0.87 | 0.88 | 1.50 |  |
| O-02-03 | 0.18 | 0.13 | 0.16 | 0.19 | 0.00 | 1.88 | 1.60 | 1.01 |  |
| O-02-02 | 0.15 | 0.16 | 0.20 | 0.22 | 0.12 | 0.00 | 4.63 | 0.86 |  |
| O-02-01 | 0.17 | 0.19 | 0.22 | 0.22 | 0.14 | 0.05 | 0.00 | 0.93 |  |
| O-01-01 | 0.22 | 0.24 | 0.28 | 0.14 | 0.20 | 0.23 | 0.23 | 0.00 |  |
