# Supplementary material for: Population Structure and Genetic Diversity Within the Endangered Species Pityopsis ruthii (Asteraceae)
Source: Front Plant Sci. 2018 Jul 11;9:943. doi: 10.3389/fpls.2018.00943 (PMC6050971; doi:10.3389/fpls.2018.00943)
Supplement: TABLE S4 — Characterization of seven chloroplast microsatellite markers from Pityopsis ruthii. [file Table_4.DOCX]

| **Table S4.** Characterization of seven chloroplast microsatellite markers from *Pityopsis ruthii.* | | | | | | | | | |
| --- | --- | --- | --- | --- | --- | --- | --- | --- | --- |
| GenBank accession | Locus | Primer sequences (5’-3’) | Repeat motif | N | N_a_ | N_e_ | I | h | uh |
| KX553852 | cpPR002 | F:ACTCACTAAGCCGGGATCACT  R:GGAACCGGGGAAAGTATACAG | (T)_9_ | 17 | 3.46 | 2.48 | 0.96 | 0.54 | 0.59 |
| KX553853 | cpPR004 | F: ACCGATCCTTGTTTACCAACC  R: TCTCGAGAAACAAGTGGGCTA | (GAA)_3_ | 5 | 2.61 | 1.79 | 0.64 | 0.38 | 0.40 |
| KX553854 | cpPR005 | F: ATTCGGCAGATTTTGATTCCT  R: AAAACCCCTTCCCAAACTGTA | (T)_12_ | 5 | 3.00 | 1.90 | 0.76 | 0.43 | 0.47 |
| KX553855 | cpPR006 | F: ATTGAATTGGGTCCAGGAATC  R:GCAATGAGATCGTTAAATGGAA | (T)_8_ | 12 | 3.21 | 2.35 | 0.85 | 0.49 | 0.52 |
| KX553856 | cpPR010 | F: AATGGACGATTCCATCGATTA  R: TGAACAAACTCGACAAATGG | (AG)_4_ | 16 | 3.91 | 2.52 | 1.00 | 0.54 | 0.60 |
| KX553857 | cpPR011 | F:CAAAATTTCTTGATTCCCATACA  R: TTTAGGCAGAATACCATCACCT | (CAG)_3_ | 15 | 3.27 | 2.04 | 0.75 | 0.40 | 0.44 |
| KX553858 | cpPR019 | F: GCGTATTGATTTGACCCCATA  R: TTGCGAAAACTTCTGGATAGG | (A)_9_ | 8 | 3.12 | 2.07 | 0.77 | 0.44 | 0.47 |
| Number of alleles (N), allele frequency (N_a_), number of effective alleles (N_e_), Shannon’s information index (I), diversity (h), and unbiased diversity (uh) | | | | | | | | | |
