# Supplementary material for: Population Structure and Genetic Diversity Within the Endangered Species Pityopsis ruthii (Asteraceae)
Source: Front Plant Sci. 2018 Jul 11;9:943. doi: 10.3389/fpls.2018.00943 (PMC6050971; doi:10.3389/fpls.2018.00943)
Supplement: TABLE S5 — Wilcoxon tests for recent bottlenecks using the program BOTTLENECK for Pityopsis ruthii sampling sites. The P-values are reported for the infinite allele model (IAM), two-phase mutational model (TPM), and stepwise mutational model (SMM). [file Table_5.DOCX]

| **Table S5.** Wilcoxon tests for recent bottlenecks using the program BOTTLENECK for *Pityopsis ruthii* subpopulations. The *P* values are reported for the infinite allele model (IAM), two-phase mutational model (TPM), and stepwise mutational model (SMM). | | | | | |
| --- | --- | --- | --- | --- | --- |
| River | Structure cluster | IAM | TPM | SMM | Distribution |
| Hiwassee | 1 (yellow) | 0.001 | 0.008 | 0.001 | Normal |
|  | 2 (blue) | <0.001 | 0.910 | 0.064 | Normal |
|  | 3 (green) | <0.001 | 0.093 | 0.001 | Normal |
|  | 4 (red) | 0.034 | 0.001 | <0.001 | Normal |
| Ocoee | 1 (red) | 0.008 | 0.470 | 0.077 | Normal |
|  | 2 (blue) | 0.017 | 0.380 | 0.850 | Normal |
|  | 3 (green) | 0.340 | 0.569 | 0.151 | Normal |
